# Pancytopenia in a Patient with Rendu-Osler-Weber Syndrome and Uncommon Vascular Abnormalities

**DOI:** 10.1155/2016/3016402

**Published:** 2016-10-10

**Authors:** Nicolò Binello, Antonio Gasbarrini, Eleonora Gaetani

**Affiliations:** Department of Internal Medicine, Agostino Gemelli University Hospital, Catholic University of Sacred Heart, Rome, Italy

## Abstract

Rendu-Osler-Weber syndrome, or hereditary hemorrhagic teleangiectasia (HHT), is a rare autosomal dominant vascular disorder, characterized by multiple mucocutaneous teleangiectases with recurrent nasal and gastrointestinal bleedings and/or solid-organ arteriovenous shunts. We describe the first case to our knowledge of pancytopenia in a 53-year-old patient, with a known history of HHT and recurrent nasal and gastrointestinal bleedings, who was found to have a major splenic artery aneurysm and other uncommon vascular abnormalities. In the absence of other evident causes of pancytopenia, hypersplenism was diagnosed. The patient underwent coil embolization of the splenic artery aneurysm, followed by rapid and sustained increase of white blood cell and platelet count. Splenic artery aneurysms are extremely uncommon in HHT as only anecdotal cases have been reported to date. However, we believe that the aneurysm critically contributed to the progression of splenomegaly and the development of pancytopenia.

## 1. Introduction

Rendu-Osler-Weber syndrome, or hereditary hemorrhagic teleangiectasia (HHT), is a rare autosomal dominant vascular disorder, mostly occurring as a result of a mutation of one of two genes involved in the TGFbeta/BMP signaling pathway (*ENG*,* ACVRL1*). HHT is characterized by multiple mucocutaneous teleangiectases with recurrent nasal and gastrointestinal bleedings and/or solid-organ arteriovenous shunts, including arteriovenous malformations (AVMs) or fistulas (AVFs), with varying degrees of penetrance and expression. We describe the first case to our knowledge of pancytopenia in a patient affected by HHT with a splenic artery aneurysm and other uncommon vascular abnormalities.

## 2. Case Presentation

**Figure 1:**
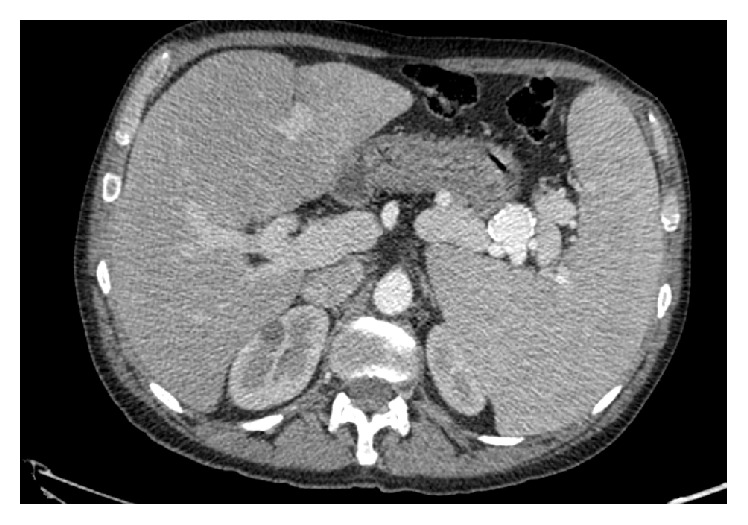
CT scan of the abdomen using IV contrast, performed before aneurysm embolization. A markedly enlarged spleen and a saccular aneurysm of the splenic artery with calcified walls are seen. A significant portal vein dilation with an increased splenoportal axis calibre is also present.

A 53-year-old man, with a known history of HHT and recurrent nasal and gastrointestinal bleedings, was admitted to our hospital because of the recent onset of pancytopenia. No evidence of pulmonary, cerebral, or hepatic vascular shunts, including AVMs, was demonstrated on prior follow-up contrast-enhanced CT scans. One month before admission, the patient's hemoglobin level was 4.8 g/dL (MCV 75.6.2 fL, MCH 18.5 pg), WBC count was 1.970/mm^3^, and platelets were 103.000/mm^3^. At admission, the patient was still pancytopenic (Hb 3.9 g/dL, WBC 2.810/mm^3^, and PLT 131.000/mm^3^). The remaining of the chemical workup was normal. Contrast-enhanced CT of the abdomen revealed a markedly enlarged spleen (17,5 cm), a splenic artery saccular aneurysm with calcified walls (28 mm) (Figure 1), and a grossly dilated portal vein (26 mm) with an increased splenoportal axis calibre. No evidence of hepatic AVMs was found, although the liver was mildly enlarged and accessory right dorsal hepatic veins were detected. Multiple vascular variants were also found, including the left hepatic artery originating from the left gastric artery and the right hepatic artery from the superior mesenteric artery. In the absence of other evident causes of pancytopenia, hypersplenism was diagnosed. The patient underwent coil embolization of the splenic artery aneurysm, followed by rapid increase of white blood cell and platelet count. During the hospital stay, single-balloon push-and-pull enteroscopy was also performed, and multiple bleeding duodenal and jejunal angiodysplasias were controlled by Argon-plasma coagulation. After six days, hemoglobin level rose to 8.2 g/dL, WBC count was 13.540/mm^3^, and platelets were 321.000/mm^3^. The patient was finally discharged and addressed to our outpatient follow-up.

## 3. Discussion

Pancytopenia is extremely rare in HHT patients. To date, only one case was described, where hypersplenism and portal hypertension were the result of multiple hepatic teleangiectases [1]. In HHT, liver involvement is usually characterized by vascular malformations, including arteriovenous, arterioportal, and portovenous shunts resulting into portal hypertension, hepatic teleangiectases, abnormal perfusion areas, or confluent vascular masses within the parenchyma [2]. In our patient, indirect signs of portal hypertension, including splenomegaly and increased portal vein diameter, were clearly demonstrated on contrast-enhanced CT. However, vascular shunts were not identified on contrast-enhanced CT nor was present a concomitant disease accounting for portal hypertension. Although microscopic vascular shunts can be missed radiographically [3], the presence of accessory hepatic veins might have played a crucial role in the development of portal hypertension.

Furthermore, this is the first ever reported case of HHT to our knowledge where a splenic artery aneurysm was identified in a pancytopenic patient. Splenic artery aneurysms are extremely uncommon in HHT and only anecdotal cases have been reported to date [4, 5]. In this case the splenic artery aneurysm may have developed as a consequence of portal hypertension and congestive splenomegaly or, alternatively, might constitute a result of the vascular dysplastic tendency of the patient. Arterial walls in HHT are usually normal and show no tendency to aneurysms, in absence of shunts, suggesting the second hypothesis is less likely. Yet, it can be speculated that the uncommon vascular abnormalities as well as the splenic artery aneurysm detected on CT could result from one or more undetermined predisposing factors that might not be necessarily correlated with HHT.

However, we believe that the aneurysm critically contributed to the progression of splenomegaly and the development of pancytopenia. Clinically significant portal hypertension in HHT, regardless of the cause, usually results in ascites or gastroesophageal varices [3]. In our patient no radiographic evidence of ascites nor endoscopic signs of varices or portal hypertensive gastropathy were demonstrated. Furthermore, coil embolization of the splenic artery aneurysm, decreasing the arterial flow toward the spleen, was followed by a rapid and sustained increase of both WBC and platelets.

## Abbreviations

ACVRL1: Activin receptor-like kinase 1
AVM: Arteriovenous malformation
AVF: Arteriovenous fistula
CT: Computed tomography
ENG: Endoglin
Hb: Hemoglobin
HHT: Hereditary hemorrhagic teleangiectasia
MCV: Mean corpuscular volume
MCH: Mean cell hemoglobin
PLT: Platelet
TGF: Transforming growth factor
WBC: White blood cell.

## References

[1] Tanvetyanon T., Nand S. (2004). Pancytopenia in hereditary haemorrhagic telangiectasia. *British Journal of Haematology*.

[2] Sabbà C., Pompili M. (2008). Review article: the hepatic manifestations of hereditary haemorrhagic telangiectasia. *Alimentary Pharmacology and Therapeutics*.

[3] Garcia-Tsao G. (2007). Liver involvement in hereditary hemorrhagic telangiectasia (HHT). *Journal of Hepatology*.

[4] Chou Y. H., Tiu C. M., Hsu C. C. (2000). Hereditary haemorrhagic telangiectasia: hepatic lesions demonstrated with colour Doppler and power Doppler sonography. *European Journal of Radiology*.

[5] Delvi M. B., Khan-Ghori S., Al-Salman M. M. S., Takrouri M. S. M. (2004). Osler-weber-rendu disease—unexpected complication following excision of spleenic aneurysm—a case report. *Middle East Journal of Anesthesiology*.

